# The Bacterial Species *Campylobacter jejuni* Induce Diverse Innate Immune Responses in Human and Avian Intestinal Epithelial Cells

**DOI:** 10.3389/fmicb.2017.01840

**Published:** 2017-09-29

**Authors:** Daniel A. John, Lisa K. Williams, Venkateswarlu Kanamarlapudi, Thomas J. Humphrey, Thomas S. Wilkinson

**Affiliations:** ^1^Microbiology and Infectious Disease, Swansea University Medical School, Institute of Life Science, Swansea University, Swansea, United Kingdom; ^2^Cellular Biology, Swansea University Medical School, Institute of Life Science, Swansea University, Swansea, United Kingdom

**Keywords:** *Campylobacter jejuni*, human and avian epithelial cells, IL-8, CXCLi1/CXCLi2, invasion, signaling, endocytosis

## Abstract

*Campylobacter* remain the major cause of human gastroenteritis in the Developed World causing a significant burden to health services. *Campylobacter* are pathogens in humans and chickens, although differences in mechanistic understanding are incomplete, in part because phenotypic strain diversity creates inconsistent findings. Here, we took *Campylobacter jejuni* isolates (*n* = 100) from multi-locus sequence typed collections to assess their pathogenic diversity, through their inflammatory, cytotoxicity, adhesion, invasion and signaling responses in a high-throughput model using avian and human intestinal epithelial cells. *C. jejuni* induced IL-8 and CXCLi1/2 in human and avian epithelial cells, respectively, in a MAP kinase-dependent manner. In contrast, IL-10 responses in both cell types were PI 3-kinase/Akt-dependent. *C. jejuni* strains showed diverse levels of invasion with high invasion dependent on MAP kinase signaling in both cell lines. *C. jejuni* induced diverse cytotoxic responses in both cell lines with *cdt*-positive isolates showing significantly higher toxicity. Blockade of endocytic pathways suggested that invasion by *C. jejuni* was clathrin- and dynamin-dependent but caveolae- independent in both cells. In contrast, IL-8 (and CXCLi1/2) production was dependent on clathrin, dynamin, and caveolae. This study is important because of its scale, and the data produced, suggesting that avian and human epithelial cells use similar innate immune pathways where the magnitude of the response is determined by the phenotypic diversity of the *Campylobacter* species.

## Introduction

*Campylobacter* is a leading cause of bacterial food-borne diarrhoeal disease worldwide, with symptoms ranging from mild to serious infections, which can result in permanent neurological damage; especially in elderly people (Silva et al., 2011). It is frequently found in poultry and chicken is an important source of *Campylobacter* infection. In the United Kingdom alone, *Campylobacter* is estimated to cause up to 700000 cases of infection and more than 100 deaths each year. *Campylobacter* infection costs the United Kingdom economy at least £900 million per year (DEFRA, 2012). In addition, *Campylobacter jejuni* is the most common species to cause a rare neuromuscular paralysis known as Guillain–Barré syndrome (Parkhill et al., 2000).

*Campylobacter jejuni* is pathogenic in humans and avian hosts although mechanistic understanding of differences is incomplete (Byrne et al., 2007; Jennings et al., 2011; Williams et al., 2013; Humphrey et al., 2014). Despite this and in limited isolates (such as M1, NCTC 11168, 13126, NCTC 12744) strain dependency is particularly well documented. Thus, individual *C. jejuni* genotypes have been shown to produce their own unique infection rates and *in vivo* behaviors in chickens when taken from the two major MLST clonal complexes (CC), CC-45 and CC-21 (Chaloner et al., 2014). This heterogeneity has also identified strains with an invasive phenotype that lead to extra-intestinal spread (Humphrey et al., 2015), and have been implicated in recent outbreaks (Harrison et al., 2013; Edwards et al., 2014). However, the differences between *C. jejuni* strains which cause invasive disease and those which remain localized in the gut are poorly understood. One recent explanation suggests that dysregulation of cytokine production leading to an over-exuberant pro-inflammatory response leads to gut damage and bacterial invasion (Humphrey et al., 2014). However, there is a relative paucity of data regarding the ability of individual *C. jejuni*, across the wide spectrum of different strains, to cause inflammation.

Genes important for *C. jejuni* virulence are associated with motility, adhesion, invasion and toxin production (**Table 1**). *C. jejuni* is a highly motile organism with bipolar flagella and motility is very important for colonization and infection in chickens and other animals (Guerry, 2007). Genes involved in motility include *flaA, flaB*, and *flaC*. The *flaA* gene is also important for invasion of epithelial cells, and is responsible for adherence and colonization by *C. jejuni* in the gastrointestinal tract (Guerry, 2007). In addition, flagella may help *C. jejuni* invasion mechanisms by serving as export apparatus in the secretion of non-flagellar proteins (Konkel et al., 2004), including the ability to deliver *flaC* and *Campylobacter* invasion antigen *(cia)* into the cell’s cytoplasm (Konkel et al., 2004). *CiaC* is required for bacterial invasion into host cells whereas *ciaI* has been reported to be required for intracellular survival of *C. jejuni* after invasion (Buelow et al., 2011; Eucker and Konkel, 2012). The ‘invasion associated protein’ is encoded by *iamA* and its exact role in this process is still unclear (Rivera-Amill et al., 2001). *HtrA*, a serine protease, may act as a chaperone protein, which affects folding of adhesins (Bæk et al., 2011). One of the main toxins produced by *C. jejuni* is cytolethal distending toxin (CDT), which causes direct DNA damage leading to the activation of DNA damage checkpoint pathways, resulting in cell death (Lee et al., 2003). CDT consists of three protein subunits (CdtA, CdtB, and CdtC), which are encoded by genes *cdtA, cdtB*, and *cdtC*. The expression of all three genes is required in order to produce an active form of CDT (Pickett et al., 1996).

**Table 1:** Presence and absence of important virulence factors in *Campylobacter jejuni* isolates used in this study.

Gene	Presence %	Absence %
**Motility**		
flaA/flaB	28.95	71.05
flaC	96.72	3.28
flgS	95.39	4.61
flgR	95.39	4.61
fliA	96.72	3.28
**Adhesion**		
cadF	96.72	3.28
pldA	93.42	6.58
peb1A	97.36	2.64
peb3	82.23	17.77
peb4	96.05	3.95
**Invasion**		
ciaB	94.07	5.93
htrA	97.37	2.63
iamA	96.05	3.95
iamB	96.71	3.29
**Toxicity**		
cdtA	86.85	13.15
cdtB	91.45	8.55
cdtC	88.82	11.18
**Misc**		
porA	96.05	3.95
fcl	48.68	51.32
hddC	14.47	85.53
rfbC	51.97	48.03
cj0794	65.78	34.22
cj0859c	46.71	53.29

Understanding the mechanisms behind *Campylobacter* interaction with the host has focussed attention on human intestinal epithelial cells (e.g., HT-29, T84, and CaCo-2) and has shown that bacterial internalization is very important in *C. jejuni* pathogenesis (Jin et al., 2003; MacCallum et al., 2006; Byrne et al., 2007; Larson et al., 2008; Friis et al., 2009; Li et al., 2011). *C. jejuni* invades intestinal epithelial cells in a microtubule-, microfilament- and caveolin-dependent manner with a distinct cell type specificity (Oelschlaeger et al., 1993; Byrne et al., 2007; Larson et al., 2008; Watson and Galán, 2008). Invasion of human intestinal epithelial cells by *C. jejuni* activates numerous downstream signaling pathways, including the MAP kinases, ERK and p38, leading to the production of the pro-inflammatory cytokine interleukin-8 (IL-8) (Hickey et al., 2000; Jin et al., 2003; Li et al., 2011) and the anti-inflammatory cytokine IL-10 in human systems (Li et al., 2011). Indeed, a relationship between IL-8 production and *C. jejuni* invasion has been previously proposed in human cells (Li et al., 2016). Whether similar responses are observed in avian epithelial cells is poorly understood and is complicated by the presence of two IL-8 orthologs, IL-8like1 (CXCLi1) and IL-8like2 (CXCLi2), which are both induced by *Campylobacter* (Larson et al., 2008).

No studies have investigated whether similar mechanisms exist across collections of *C. jejuni* strains that define the species and have been isolated from relevant environmental, veterinary or clinical sources. In addition, previous work alluded to above in human cells, and the very limited work in chicken epithelial cells, do not represent the diversity across the *Campylobacter* species (<8 strains). In this work, we investigated the response of 100 *C. jejuni* strains that have been characterized previously at the genome level (Sheppard et al., 2013) by investigating their inflammatory (cytokine), adhesion, invasion, toxicity and signalling responses in 8E11 (avian) intestinal epithelial cells and compared these responses with human intestinal epithelial cells (HT-29). The host responses measured here suggest that avian and human epithelial cells share common mechanisms to combat *C. jejuni* but there is exceptional phenotypic diversity in the bacterial population.

## Materials and Methods

### Bacterial Strains, Genomes, and Culture Conditions

**FIGURE 1:**
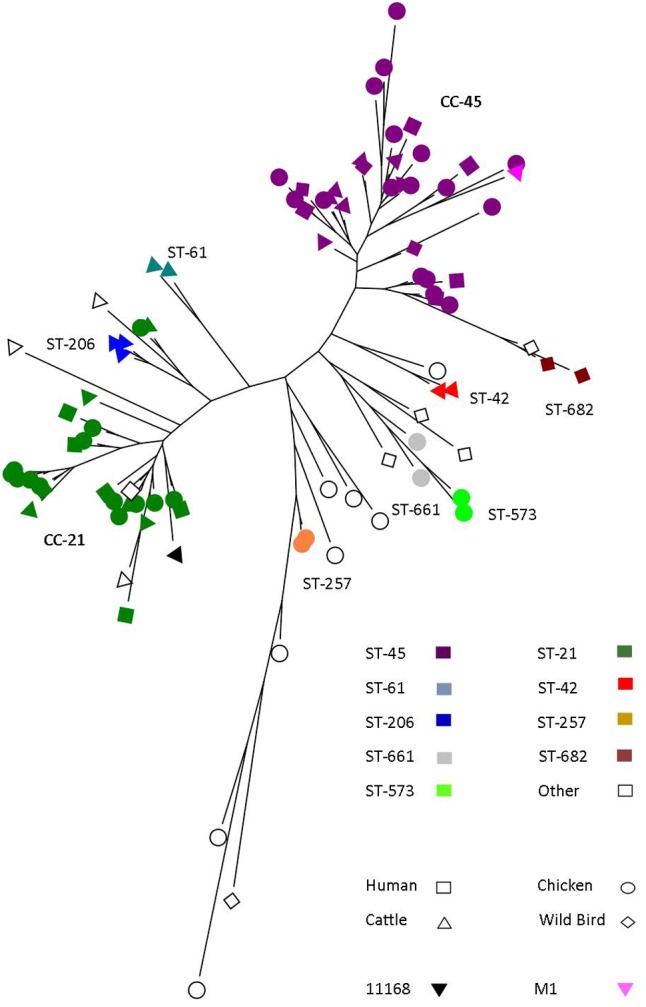
*Campylobacter jejuni* strains used within the study. A collection of 100 *C. jejuni* strains from a variety of sources and sequence types that represent the species.

A collection of 100 fully sequenced isolates of *C. jejuni* from a variety of sources and sequence types were used in this study and had been characterized previously at the genomic level (Sheppard et al., 2011, 2013) (**Figure 1** and **Table 2**). In brief, sequences were annotated using Prokka (Seemann, 2014). The resulting assemblies were used for calculations in Roary (Page et al., 2015) to create a pan-genome. Then, MEGA 6 (Tamura et al., 2013) was used to visualize the resulting data on a phylogenetic tree. *C. jejuni* strains were cultured under microaerobic conditions (5% O_2_, 10% CO_2_, 85% N_2_) on *Campylobacter* blood free selective medium (mCCDA; Oxoid) plates at 42°C (Davis and DiRita, 2008). One colony of cultured *C. jejuni* was then inoculated into Muller-Hinton (MH) broth and grown for 24 h at 42°C before being used in downstream assays.

**Table 2:** List of 100 strains used in this study.

Isolate	Species	Clonal complex	Source	IL-8
CAMP45	*C. jejuni*	ST-45	Chicken	
CAMP61	*C. jejuni*	ST-61	Cattle	
CampsClin11	*C. jejuni*	ST-45	Human	High
CampsClin45	*C. jejuni*	ST-45	Human	High
CampsClin262	*C. jejuni*	ST-21	Human	
CampsClin583	*C. jejuni*	ST-45	Human	High
CampsClin266	*C. jejuni*	ST-21	Human	
CampsClin883	*C. jejuni*	ST-21	Human	High
CampsClin1003	*C. jejuni*	ST-45	Human	
Chick2219	*C. jejuni*	ST-45	Chicken	
Chicka21	*C. jejuni*	ST-21	Chicken	
Cow55	*C. jejuni*	–	Cattle	
Cow42	*C. jejuni*	ST-42	Cattle	
Chick2253	*C. jejuni*	–	Chicken	
Chick594	*C. jejuni*	ST-45	Chicken	
Cow2673	*C. jejuni*	–	Cattle	
Cow2674	*C. jejuni*	ST-21	Cattle	
Cow206	*C. jejuni*	ST-206	Cattle	
Cow38	*C. jejuni*	ST-48	Cattle	
Cow190	*C. jejuni*	–	Cattle	
Cow334	*C. jejuni*	ST-45	Cattle	
Chicka45	*C. jejuni*	–	Chicken	
Chick267	*C. jejuni*	ST-283	Chicken	
CampsClin230	*C. jejuni*	ST-45	Human	
Cowa45	*C. jejuni*	ST-45	Chicken	
Chick2213	*C. jejuni*	ST-45	Chicken	
Cow518	*C. jejuni*	ST-21	Cattle	High
CampsClin53	*C. jejuni*	ST-21	Human	
Cow58	*C. jejuni*	–	Cattle	
Cowa21	*C. jejuni*	ST-21	Cattle	
Chickc21	*C. jejuni*	ST-21	Chicken	
Chick25	*C. jejuni*	ST-661	Chicken	
Chick104	*C. jejuni*	ST-21	Chicken	
Chick353	*C. jejuni*	ST-353	Chicken	
Chickb354	*C. jejuni*	ST-354	Chicken	
Chick573	*C. jejuni*	ST-573	Chicken	
Chick2568	*C. jejuni*	ST-661	Chicken	
Chickc45	*C. jejuni*	ST-45	Chicken	Low
Chick19	*C. jejuni*	ST-21	Chicken	Low
Chick50	*C. jejuni*	ST-21	Chicken	High
Chick53	*C. jejuni*	ST-21	Chicken	Low
Chick262	*C. jejuni*	ST-21	Chicken	
Chick266	*C. jejuni*	ST-21	Chicken	
Chick861	*C. jejuni*	–	Chicken	
Chick1086	*C. jejuni*	ST-21	Chicken	
Chick1360	*C. jejuni*	ST-21	Chicken	
Chick11	*C. jejuni*	ST-45	Chicken	
Chick137	*C. jejuni*	ST-257	Chicken	High
Chick1003	*C. jejuni*	ST-45	Chicken	
Chick2048	*C. jejuni*	ST-45	Chicken	
Chick2197	*C. jejuni*	ST-354	Chicken	
Chick2223	*C. jejuni*	ST-45	Chicken	Low
Cow3583	*C. jejuni*	ST-42	Cattle	Low
Cow618	*C. jejuni*	ST-61	Cattle	Low
Cow237	*C. jejuni*	ST-206	Cattle	High
Cow270	*C. jejuni*	ST-403	Cattle	Low
Cowb21	*C. jejuni*	ST-21	Cattle	
Cowb45	*C. jejuni*	ST-45	Cattle	
Cowc45	*C. jejuni*	ST-45	Cattle	
Cowd45	*C. jejuni*	ST-45	Cattle	
Cow53	*C. jejuni*	ST-21	Cattle	
Cow104	*C. jejuni*	ST-21	Cattle	Low
Cow3189	*C. jejuni*	–	Cattle	High
Cow3201	*C. jejuni*	ST-21	Cattle	
Cow3205	*C. jejuni*	ST-206	Cattle	
Cow137	*C. jejuni*	ST-45	Cattle	
Cow230	*C. jejuni*	–	Cattle	
Cow583	*C. jejuni*	ST-45	Cattle	
Cow3207	*C. jejuni*	ST-45	Cattle	High
Cow3214	*C. jejuni*	ST-45	Cattle	
Chick354	*C. jejuni*	ST-257	Chicken	
Chick51	*C. jejuni*	ST-443	Chicken	
Chick1079	*C. jejuni*	ST-573	Chicken	Low
Chick574	*C. jejuni*	ST-574	Chicken	
Chick814	*C. jejuni*	ST-661	Chicken	
Chickb21	*C. jejuni*	ST-21	Chicken	
Chickb45	*C. jejuni*	ST-45	Chicken	
Chickd45	*C. jejuni*	ST-45	Chicken	High
Chick883	*C. jejuni*	ST-21	Chicken	
Chick230	*C. jejuni*	ST-45	Chicken	
Chick2663	*C. jejuni*	ST-45	Chicken	
CampsClin21	*C. jejuni*	–	Human	High
OxClina21	*C. jejuni*	ST-21	Human	
OxClinb21	*C. jejuni*	ST-45	Human	Low
OxClina45	*C. jejuni*	ST-45	Human	High
OxClinb45	*C. jejuni*	ST-21	Human	High
Starling177	*C. jejuni*	ST-177	Starling	Low
Starling682	*C. jejuni*	ST-682	Starling	Low
Starling45	*C. jejuni*	ST-45	Starling	
Starling1020	*C. jejuni*	ST-682	Starling	Low
Goose1033	*C. jejuni*	ST-1034	Goose	High
Goose702	*C. jejuni*	–	Goose	Low
Goose137	*C. jejuni*	ST-45	Goose	Low
Goose696	*C. jejuni*	ST-1332	Goose	Low
Duck702	*C. jejuni*	ST-702	Duck	
Duck45	*C. jejuni*	ST-45	Duck	Low
CAMP2381	*C. jejuni*	–	Environmental waters	
NCTC11168	*C. jejuni*	ST-21	Human	High
M1	*C. jejuni*	ST-45	Human	High

### Motility Assays

Two milliliters of MH medium supplemented with 0.4% agar was aliquoted to each well of a 6-well plate and allowed to solidify. Then, 2 μl of *C. jejuni* suspensions (0.1 OD_600_) were added to the center of a well, and the plate incubated at 37°C under microaerobic conditions for 48 h. Relative motility of each bacterial strain was determined by measuring the diameter of the migration zone.

### Growth Assays

*Campylobacter jejuni* growth was measured using a semi-quantitative assay in Nunc 96-well tissue culture plates (Pascoe et al., 2015). Briefly, *C. jejuni* strains were grown overnight in MH liquid medium at 37°C under microaerobic conditions (5% O_2_, 10% CO_2_, 85% N_2_) and diluted using MH to 0.1 OD_600_. Five microlitres of the diluted bacterial suspension were inoculated into 200 μl of fresh MH and bacterial growth monitored in real-time over 48 h at 37 and 42°C in a FLUOstar OMEGA plate reader (BMG LabTech, Bucks United Kingdom) equipped with an atmospheric control unit to maintain a microaerobic atmosphere (5% O_2_, 10% CO_2_, 85% N_2_). Spectrophotometric measurements were taken at OD_600_ every 60 min and the average of at least three replicates was calculated.

### Culture of Human and Chicken Epithelial Cells

Human colon epithelial adenocarcinoma cells (HT-29) were grown in McCoy’s 5A (Modified) medium supplemented with L-glutamine (5 mM), Penicillin (10,000 U/ml), Streptomycin (10,000 U/ml) (G/P/S) and 10% foetal bovine serum (FBS). Chicken epithelial cells (MM-CHiC clone, 8E11 (Micromol, Germany) were maintained in Dulbecco’s modified eagle medium/nutrient mixture F-12 (DMEM/F-12) with G/P/S and 10% FBS. Cultures were maintained in T75 flasks at 37°C in a 5% CO_2_ incubator.

### Epithelial Cell Viability Assay

This was assessed using the AlamarBlue Reagent (Thermo Fisher Scientific) according to manufacturer’s instructions. AlamarBlue cell viability reagent functions as a cell health indicator using the reducing power of living cells. Viable cells are able to continuously convert resazurin, the active ingredient in alamarblue, to resorufin and so increasing the overall fluorescence and color of the media. Results are presented as a percentage reduction in cell viability. Briefly; 5 μl of AlamarBlue reagent was added to each well of a 96-well plate containing HT-29 or 8E11 cells infected with *C. jejuni* in 50 μl of conditioned medium. Plates were incubated for 4 h at 37°C and absorbance was measured at OD_570_, and OD_600_.

### *C. jejuni*-Induced Cytokine Production

#### Infection of Epithelial Cell Monolayers

Cell monolayers, containing 3.5 × 10^5^ cells/well, grown in a 24-well tissue culture plate were infected with 5 × 10^6^ cfu of *C. jejuni* for 24 h at 37°C in a 5% CO_2_ atmosphere to allow the bacteria to adhere to and invade the host cells.

#### RNA Isolation from Infected Epithelial Cells

Following the infection period, total RNA was isolated from HT-29 human or 8E11 chicken intestinal epithelial cells grown in a 24 well plate using the method provided with the Promega SV total RNA isolation kit (Promega, Southampton, United Kingdom). Total RNA was quantified using a NanoDrop (Thermo scientific, Loughborough, United Kingdom) and run through a 0.7% agarose gel using a 1 kb and 100 bp ladders to confirm integrity.

#### Quantitative PCR of Cytokine RNA Transcripts from Infected Monolayers

One microgram of total RNA was converted to cDNA using an iScript kit (Bio-Rad). Quantitative PCR was used to amplify the gene of interest and the housekeeping gene. Each reaction (25 μl) contained 12.5 μl 2X Sensimix SYBR buffer (Bioline), 0.5 μl each primer (25 μM), 9.5 μl purified water and 2 μl cDNA. The qPCR conditions were as follows; 10 min at 95°C, then 50 cycles with denaturing for 15 s at 95°C, annealing for 15 s at temperatures specific to primers sets (**Table 3**) and synthesis at 72°C. Reactions were performed in an iCycler (Bio-Rad). Primer efficiency was measured using total RNA from epithelial cells infected with a reference *C. jejuni* isolate (NCTC11168) and a dilution series up to 1/10,000. The log values of the Cycle threshold (CT) values were then taken and plotted graphically and the slopes used to calculate the efficiency. Relative transcriptional levels within distinct experiments were determined by using the 2^-ΔΔ^Ct method and β-actin as the reference housekeeping gene (Livak and Schmittgen, 2001). Primer sequences for human IL-8, IL-10, and β-actin and chicken CXCLi1/2 and β-actin were used to create primers for qPCR (**Table 3**). Target sequences were identified from the NCBI database and then the coding sequence (CDS) was used to generate forward and reverse primers using Primer3, selecting for amplicon sizes of 50–150 bases.

**Table 3:** Primer sequences used in this study.

Genbank	cDNA	bp	AA	Primer sequence	Annealing T°C	Expected size (Kb)
BC013615.1	Human IL-8	300	99	cagttttgccaaggagtgct ttggggtggaaaggtttgga	60	73
NM_205018.1	Chicken CXCLi1	315	104	cgattgaactccgatgccag cattcttgcagtgaggtccg	59	105
NM_205498.1	Chicken CXCLi2	312	103	ggatggaagagaggtgtgct ctgagccttggccataagtg	59	79
NM_000572	Human IL-10	537	178	ggcgctgtcatcgatttctt cattcttcacctgctccacg	60	63
AJ621254.1	Chicken IL-10	528	175	acatccaactgctcagctct atgctctgctgatgactggt	59	142
X00351.1	Human β-actin	1128	375	tggcatccacgaaactacct cgtacaggtctttgcggatg	60	68
L08165.1	Chicken β-actin	1128	375	aagatcattgccccacctga cctgcttgctgatccacatc	59	100

### Invasion and Adhesion

Bacterial strains were inoculated onto plates and grown in a microaerobic environment for 48 h. A colony of freshly grown culture was sub-cultured in MH broth for 24 h, as described previously. Then, 5 × 10^6^ cfu of bacterial suspension was added to the wells containing monolayers of cells in assay medium (modified McCoy’s 5A/DMEM/F-12 with L-glutamine (5 mM) and supplemented with 5% FBS) for 6 h. The rest of the broth was serially diluted in PBS and plated out onto Columbia blood agar (COLBA) plates for enumeration of *C. jejuni*. Monolayers of cells were grown in a 6-well tissue culture plate as previously discussed. For the adhesion assay, the monolayer cells incubated with bacteria were washed three times with PBS and then incubated with maximum recovery diluent for 10 min (1.5 g peptone, 8.5 g sodium chloride, per liter; final pH 7.0) to remove unbound bacteria. Plates were shaken and adhering *C. jejuni* cells were removed and serially diluted in maximum recovery diluent and plated out onto COLBA for enumeration. For the invasion assay, the monolayer cells incubated with bacteria were washed twice with PBS before 2 ml gentamicin in PBS (100 μg/ml) was added to each well and incubated at 37°C for 90 min. Time-course analysis confirmed that *Campylobacter* strains were killed between 60 and 90 min after gentamicin exposure with longer times affecting intracellular *Campylobacter* counts. Cells were washed twice with PBS before 2 ml 0.1% Triton X-100 in PBS was added to each well in order to lyse the cells. After 5 min, cell lysates were serially diluted in PBS and plated out on COLBA plates for enumeration of the invasive bacteria. This experiment was performed four times. The limit of detection was 50 CFU/ml.

### Inhibition Assays

A series of known endocytosis and signaling inhibitors were used to block cellular processes (**Table 4**). Cells were cultured as described above for infection experiments, except that HT-29 and 8E11 cells were treated with each inhibitor separately for 30 min and prior to infection with *Campylobacter* and subsequent invasion assays and RNA isolation/qPCR. Initial inhibitor concentration ranges were identified from their previous use in HT-29 epithelial cells and/or *Campylobacter* invasion studies (Wooldridge et al., 1996; Wells et al., 1998, 1999; Hickey et al., 2000; Jin et al., 2003; Fernandez de Mattos et al., 2008; Weflen et al., 2010; Colin et al., 2011; Li et al., 2011). The optimal concentrations (**Table 4**) which include, Dynasore (20 μM), filipin, (20 μM), genistein (20 μM), chlorpromazine (20 μM), LY294002 (20 μM), In solution Akt inhibitor V, Triciribine (20 μM), PD98059 (20 μM), methyl β-cytodextrin (5 μM) and cytochalasin D (5 μM) are the highest concentrations used in this study that did not result in significant decreases in toxicity using the alamar blue assay on both HT-29 and 8E11 epithelial cells.

**Table 4:** List of Inhibitors used in study.

Inhibitor	Pathway/mechanism	Reference
Dynasore (20 μM)	Dynamin – Endocytosis, Dynamin GTPase activity	Macia et al., 2006
Filipin (20 μM)	Lipid raft Caveolin pathway Endocytosis	Bonneau et al., 2010
Genistein (20 μM)	Caveolin Endocytosis, tyrosine kinase inhibitor	Akiyama et al., 1987
Chlorpromazine (20 mM)	Clathrin Endocytosis, clathrin misassembly	Lee et al., 2007
LY294002 (20 μM)	PI-3 Kinase	Chaussade et al., 2007
InSolution^TM^ Akt Inhibitor V, Triciribine (20 μM)	Akt	Karst et al., 2006
PD98059 (20 μM)	ERK/MEK	Alessi et al., 1995
Cytochalasin D	Actin polymerization	Goddette and Frieden, 1986
Methyl β-cytodextrin	Lipid rafts/extraction of cholesterol	Rodal et al., 1999

### Statistical Analysis

The non-parametric Kruskal–Wallis test, for multiple comparisons with *post hoc* Dunns test was used. Correlations were assessed using linear regression of log transformed data with a *p*-value related to the slope. Significance differences were accepted if *p* ≤ 0.05. Graphpad Prism 6.0 (San Diego, CA, United States) was used to analyze and assess differences between treatment groups.

## Results

### *C. jejuni* Strains in the Study Population

To investigate the diversity of human and avian epithelial cell innate immune responses, 100 strains of *C. jejuni* were selected from across a phylogenetic tree (**Figure 1** and **Table 2**). This included isolates from a variety of sequence types, and the major clonal complexes CC-45 and CC21 (**Figure 1**). In addition, strains were selected based on the source of the isolate and included, human, chicken, cattle, and wild-bird isolates (**Figure 1**).

### *C. jejuni* Strains Produce a Large Variation in Inflammatory Cytokine Responses

**FIGURE 2:**
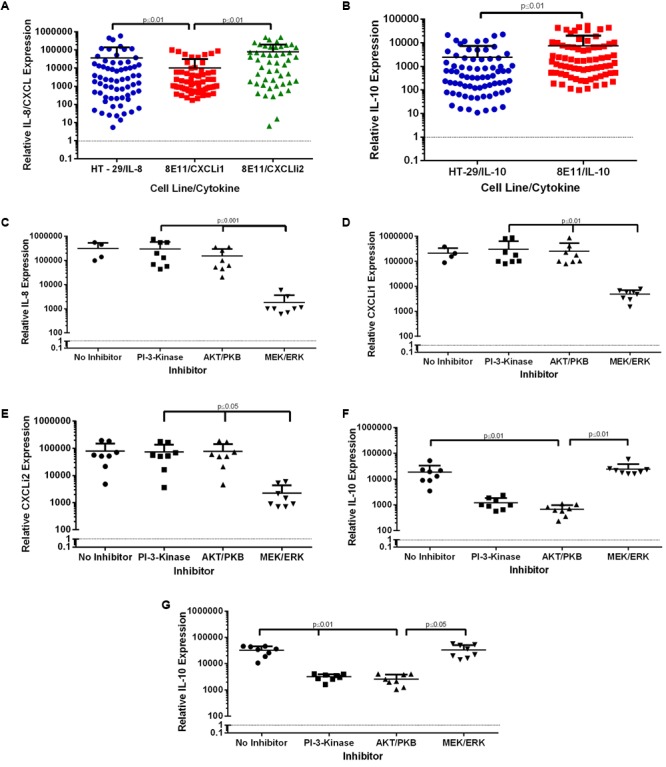
Expression of pro- and anti- inflammatory cytokines and chemokine’s in HT-29 human and 8E11 chicken epithelial cells. HT-29 and 8E11 cells were infected with *C. jejuni* for 24 h before isolation of total RNA and quantification of specific mRNA by qPCR. **(A)** Expression of IL-8/CXCLi1/CXCLi2 in both HT-29 and 8E11 cell lines **(B)** expression of IL-10 in HT-29 and 8E11 cell lines. **(C–G)** Prior to infection, cells were incubated with signaling inhibitors for up to 30 min. Then specific mRNA for IL-8/CXCLi1/CXCLi2 **(C–E)** and IL-10 **(F,G)** were measured. Each dot represents three biological replicates in one strain. Results are also expressed as mean ± SD of all strains measured. Differences were considered significant if *p* ≤ 0.05.

Inflammatory phenotype was investigated by infecting human and avian epithelial cells with the *C. jejuni* strain collection (*n* = 100). IL-8 or CXCLi1 and CXCLi2 expression in these cells showed dramatic changes compared to uninfected ones with up to 100,000-fold increases in both human and avian cells (**Figure 2A**). Despite the large variation, human IL-8 and avian CXCLi2 expression were significantly increased compared to CXCLi1 (*p* ≤ 0.01 and *p* ≤ 0.01, respectively). There was no difference between IL-8 and CXCLi2 expression. The reference strains NCTC11168 and M1 produced IL-8 and CXCLi1 responses similar to the average for the whole *C. jejuni* study population. The average *C. jejuni* induced CXCLi2 response was similar to that of the M1 but the 11168 strain-induced response was 11-fold higher. We could not identify differences in responses between sources and sequence types.

The anti-inflammatory cytokine IL-10 also displayed infection-induced increase in expression and variability compared to uninfected control (*p* ≤ 0.001) in both HT-29 and 8E11 cells. In addition, IL-10 expression was significantly increased (*p* < 0.01) in 8E11 cells compared to that in HT-29 ones (**Figure 2B**). The reference strains NCTC11168 and M1 produced IL-10 responses similar to the average for the whole *C. jejuni* study population. As above, we could not identify any difference in responses between sources and sequence types.

We then chose eight strains of *C. jejuni* that produced the strongest cytokine responses and investigated whether they affect common signaling pathways (PI 3-kinase/Akt and ERK) differently in human and avian epithelial cells (**Figures 2C–E**). Inhibition of signaling pathways showed that IL-8 expression in HT-29 cells and CXCLi1 and CXCLi2 in 8E11 cells were all ERK-dependent (*p* < 0.001, *p* < 0.01, and *p* < 0.05, respectively). Furthermore, PI 3-kinase and Akt pathways did not appear to be required for IL-8 or CXCLi1/2 production. In contrast, IL-10 expression in human and avian cells was dependent on PI 3-Kinase and its downstream target Akt but was independent of ERK (**Figures 2F,G**).

These results confirm that similar signaling pathways are responsible for IL-8 and IL-10 expression in human and avian epithelial cells.

### *C. jejuni* Invasion Is ERK-Dependent in Human and Avian Epithelial Cells

**FIGURE 3:**
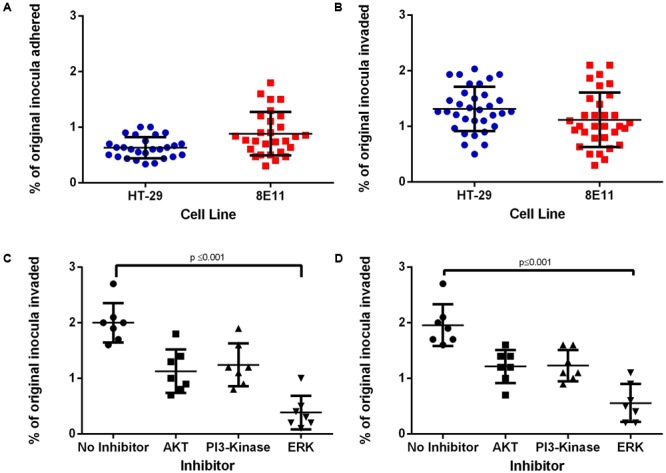
Adhesion and Invasion of *C. jejuni* into HT-29 and 8E11 cells. HT-29 and 8E11 cells were infected with *C. jejuni* for 6 h before isolation and quantification of adherent and invasive *C. jejuni*. **(A)** Adhesion of *C. jejuni* strains to HT-29 and 8E11 cells. **(B)** Invasion of *C. jejuni* into HT-29 and 8E11 cells. **(C,D)** Treatment of HT-29 and 8E11 cells with signaling inhibitors for 30 min prior to invasion assays. Each dot represents three biological replicates in one strain. Results are also expressed as mean ± SD of all strains measured. Differences were considered significant if *p* ≤ 0.05.

We investigated the ability of high and low IL-8 (or CXCLi1/2)-inducing *C. jejuni* strains (*n* = 35, final column **Table 2**) to adhere to and invade human and avian epithelial cells. No significant difference was detected in adherence to human and avian epithelial cells (**Figure 3A**) although adhesion levels to avian cells had a wider distribution. Gentamicin protection assays in both cell lines showed that all strains tested were able to invade intestinal epithelial cells in both human and avian systems (**Figure 3B**), which ranged from 1 to 3% of the starting inoculum. While each strain produced a unique invasion response no significant difference in the invasion was observed between human and avian cells. Inhibition of epithelial cell signaling pathways with ‘high’ invasive strains (*n* = 8) confirmed the role of ERK in *C. jejuni* invasion of human (**Figure 3C**) and avian (**Figure 3D**) epithelial cells (*p* ≤ 0.001 in both cases).

These results confirm the different invasion responses of individual *C. jejuni* strains despite all requiring ERK for a full invasion response.

### The *cdtA* Gene Has an Important Role in *C. jejuni*-Induced Epithelial Cell Toxicity

**FIGURE 4:**
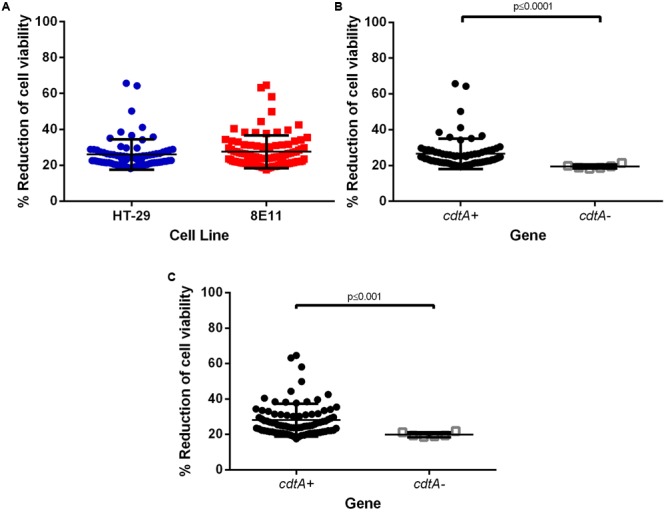
Reduction of cell viability after infection with *C. jejuni.* HT-29 and 8E11 cells were infected with *C. jejuni* for 24 h before isolation of cell supernatants for alamar blue viability assay. **(A)** Toxicity responses in HT-29 and 8E11 epithelial cells. **(B,C)** Toxicity responses organized by the presence and absence of the *cdtA* gene. Each dot represents three biological replicates in one strain. Results are also expressed as mean ± SD of all strains measured. Differences were considered significant if *p* ≤ 0.05.

*Campylobacter* invasion can compromise epithelial cell viability and we investigated toxicity responses of all *C. jejuni* strains (**Figure 4**, *n* = 100). The toxicity of *C. jejuni* for both human and avian cells showed unique profiles for each strain tested. Epithelial cells infected with any *C. jejuni* strain showed increased toxicity compared to untreated cells but only a few *C. jejuni* strains induced high toxicity responses (over 50% reduction in viability, **Figure 4A**). We could not identify differences between sources and sequence types. *cdtA*-positive strains were significantly more toxic than -negative *ones*, in both human (**Figure 4B**, *p* ≤ 0.0001) and avian epithelial cells (**Figure 4C**, *p* ≤ 0.001).

### *C. jejuni*-Induced IL-8 Production, Toxicity and Invasion of Epithelial Cells Are More Closely Correlated in Avian Cells Than in Human Ones

**FIGURE 5:**
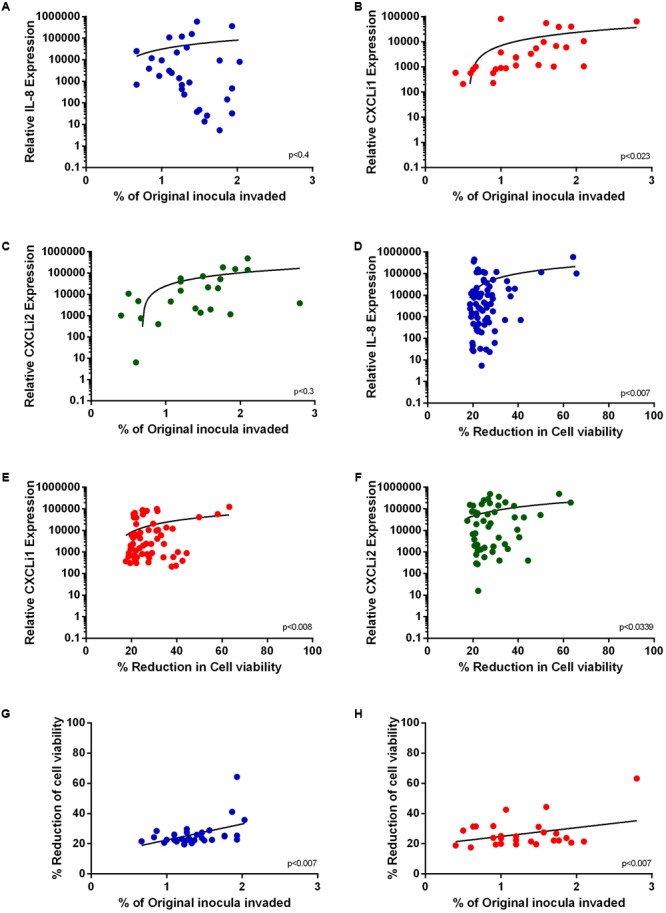
Relationships between *C. jejuni*-induced cellular invasion, toxicity and cytokine production. Phenotypic data was subjected to regression analysis. **(A)** IL-8 **(B)** CXCLi1 and **(C)** CXCLi2 correlations with *C. jejuni* invasion. **(D)** IL-8, **(E)** CXCLi1, **(F)** CXCLi2 correlations with toxicity. **(G)** HT29 cell viability correlation with invasion **(H)** 8E11 cell viability correlation with invasion.

Given that both IL-8/CXCLi1/CXCLi2 production and invasion were ERK-dependent across the collection of *C. jejuni* strains, we investigated whether correlations existed between the measured phenotypes of IL-8/CXCLi1/CXCLi2 production, invasion and toxicity. IL-8 (**Figure 5A**, *p* < 0.4) or CXCLi2 (**Figure 5C**, *p* < 0.3) expression did not correlate with *Campylobacter* invasion whereas CXCLi1 (**Figure 5B**, *p* < 0.023) expression showed strong positive correlations with invasion. IL-8 (*p* < 0.007), CXCLi1 (*p* < 0.0082), and CXCLi2 (*p* < 0.0339) all positively correlated with cell toxicity (**Figures 5D–F**). Finally, invasion and toxicity were strongly correlated in both human (*p* < 0.0078) and avian (*p* < 0.0078) cells (**Figures 5G,H**).

These correlations confirm a close interrelationship between (i) *C. jejuni*-induced cytokine expression and toxicity and (ii) between toxicity and cellular invasion.

### Endocytosis of *C. jejuni* Is Dynamin- and Clathrin-Dependent in Both Human and Avian Epithelial Cells

**FIGURE 6:**
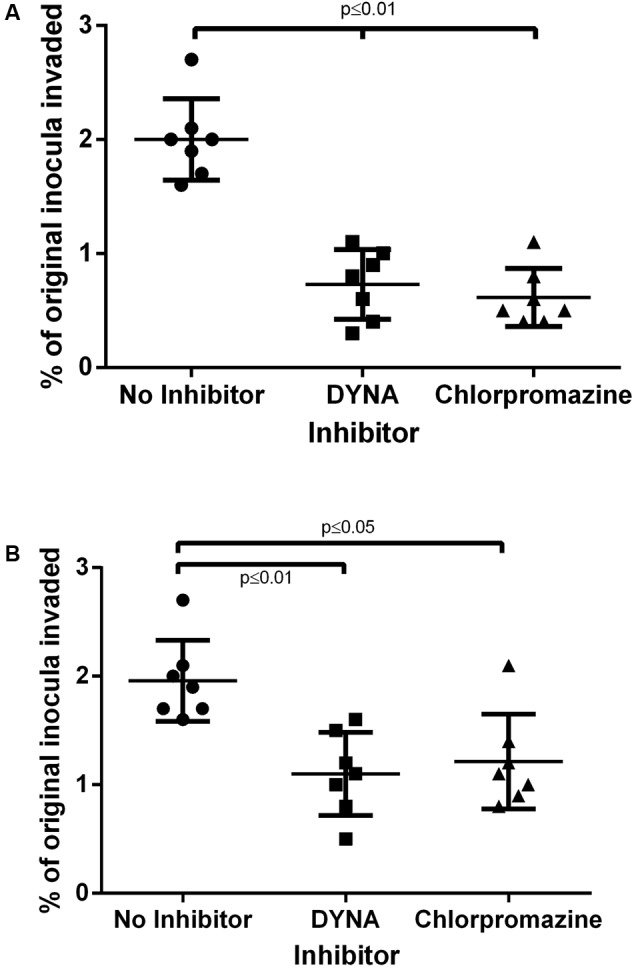
The effect of inhibition of cellular endocytosis on *C. jejuni* invasion into epithelial cells. **(A)** HT-29 and **(B)** 8E11 cells were pretreated with endocytosis inhibitors prior to infection with *C. jejuni* for 6 h and then *C. jejuni* invasion was assessed. Each dot represents three biological replicates in one strain. Results are also expressed as mean ± SD of all strains measured. Differences were considered significant if *p* ≤ 0.05.

Having confirmed the importance of ERK in downstream signaling for invasion, we next investigated upstream pathways at the cell surface important for endocytosis. Both Methyl-β-cyclodextrin and cytochalasin D completely abrogated *C. jejuni* (*n* = 31) invasion into both cell lines (**Supplementary Figure S1**) confirming the role of lipid rafts and the actin cytoskeleton, respectively. Pre-treatment of epithelial cells with a dynamin inhibitor (Dynasore) and a clathrin inhibitor (chlorpromazine) significantly reduced *C. jejuni* invasion in human (**Figure 6A**) and avian (**Figure 6B**) cells compared to the ‘no’ inhibitor control. This is confirmed by concomitant reduction in cellular toxicity in the relevant cultures (data not shown). In addition, caveolin-dependent endocytosis was tested using filipin and genistein with no consistent effect observed over triplicate experiments. These results confirm the importance of clathrin and dynamin in *C. jejuni* invasion.

### *C. jejuni*-Induced IL-8 and CXCLi1/2 Expression Is Dynamin- and Clathrin-Dependent in Human and Avian Epithelial Cells

**FIGURE 7:**
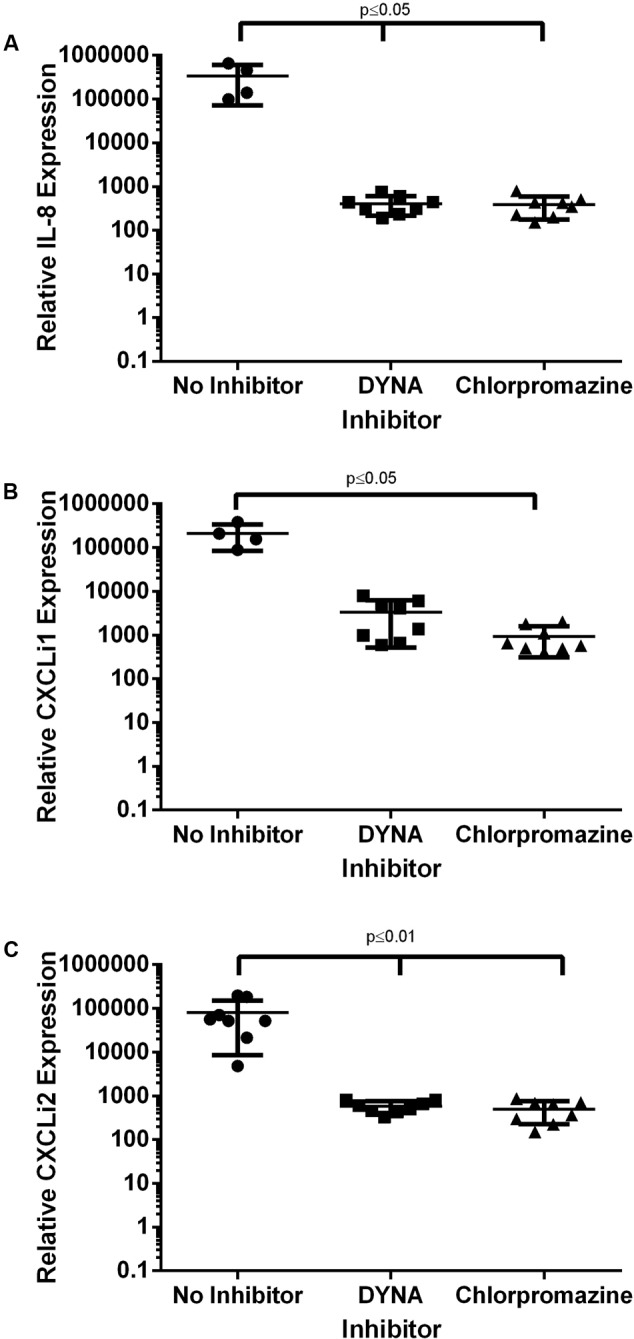
The effect of inhibition of endocytosis of cytokines cytokine expression in epithelial cells. **(A)** HT-29 and **(B,C)** 8E11 cells were treated with endocytosis inhibitors prior to infection with *C. jejuni* for 24 h. Then total RNA was isolated and specific mRNA was quantified by qPCR. Expression of **(A)** IL-8, **(B)** CXCLi1, and **(C)** CXCLi2 in epithelial cells. Each dot represents three biological replicates in one strain. Results are also expressed as mean ± SD of all strains measured. Differences were considered significant if *p* ≤ 0.05.

Cytokine expression was also determined following manipulation of endocytosis pathways. Consistent with invasion responses (**Figure 6**), IL-8 expression (**Figure 7A**) in human cells and CXCLi1 (**Figure 7B**) and CXCLi2 (**Figure 7C**) expression in avian cells was dependent on dynamin and clathrin (**Figure 7**). In contrast to invasion responses, inhibition of caveolin pathways also significantly reduced IL-8, CXCLi1 and 2 expression (data not shown). Cytokine expression could be detected in the absence of toxicity and with minimal invasion responses (**Figures 6, 7**). These results confirm the importance of clathrin, caveolin and dynamin for *C. jejuni*-induced IL-8 production.

## Discussion

The data in this paper add to the growing body of evidence that supports the idea that *C. jejuni* is a pathogen in chickens (Neill et al., 1984; Byrne et al., 2007; Jennings et al., 2011; Williams et al., 2013; Humphrey et al., 2014). Thus, *C. jejuni* induce inflammatory and toxicity responses and can also invade human and importantly avian epithelial cell lines. While there was little difference between human and avian cell responses, there was a wide range across all bacterial strains studied. We deliberately chose 100 strains of *C. jejuni* from a variety of sources (including chicken, human, cattle, and wild birds) and across sequence types to give a good representation of strains studied previously at the genomic level although we did not detect differences between these groups (Sheppard et al., 2011, 2013). Coincidentally, the reference strains M1 and NCTC11168 produced responses equivalent to the average for the whole study population. The question remains as to the source of the variation across the whole population. Interestingly, the presence of key virulence factors associated with *C. jejuni* pathogenesis was relatively high (>80%, **Table 1**) and thus differential gene expression may be responsible for the dramatic differences in epithelial cell responses. We did not measure individual virulence factor expression in this study but previous work on the transcriptome of a variety of *Campylobacter* strains showed that they are subject to complex regulation (Dugar et al., 2013).

Epithelial-derived IL-8 production is vital for early neutrophil infiltration into the gut in humans (Bennett et al., 2010) and chickens (Humphrey et al., 2014). This study confirms the importance of human IL-8 and identifies both CXCLi1 and CXCLi2 as important early chemokines induced in avian epithelial cells following *C. jejuni* infection. This is consistent with studies in chicken LMH epithelial cells (Larson et al., 2008). Interestingly, CXCLi2 (like human IL-8) was significantly higher than CXCLi1. To our knowledge this is the first time that a study using a large collection of *C. jejuni* strains has found differences in these two chicken IL-8 homologs. On a smaller scale (*n* = 6 strains), Larson et al. (2008) found the opposite effect. It is interesting to speculate that CXCLi2 (and not CXCLi1) may be the functional equivalent to IL-8 in humans. This is supported by structural data on amino acid similarity where CXCLi1 and CXCLi2 are 48 and 67%, respectively, identical to human IL-8 (Sick et al., 2000; Gupta et al., 2008).

The three cellular responses defined by ‘IL-8/CXCLi1/CXCLi2 production,’ ‘cellular toxicity,’ and ‘*Campylobacter* invasion’ are not always inextricably linked as demonstrated by the correlation curves (**Figure 5**). However, clear links were demonstrated between increased cytokine output (IL-8, CXCLi1 and 2) and ‘toxicity’ suggesting similar mechanisms of induction. Indeed, Hickey et al. (1999, 2000) suggested two mechanisms of *Campylobacter*-induced IL-8 production in human cells involving; (i) adherence and or invasion; and (ii) cdt expression. While this known link between IL-8 and invasion was demonstrated in INT407 human epithelial cells, we did not find a significant correlation between them in HT-29 cells. This was also the case for CXCLi2 production and invasion in avian cells. In contrast, a strong positive correlation was made in avian cells between CXCLi1 and invasion, again supporting an important role for these cytokines in early *Campylobacter* responses in chickens.

Numerous human cell lines have been used to study *Campylobacter* pathogenesis, including T84 (Zheng et al., 2008), INT407 (Borrmann et al., 2007), HT-29 (Bahrami et al., 2011), and CaCo-2 (Man et al., 2010) intestinal epithelial cells. There is very little data in avian intestinal cell systems. These cell lines are particularly useful for high throughput studies with many strains such as the current study. When considering the three cellular endpoints of IL-8 expression, cellular toxicity, and *Campylobacter* invasion none of these cells mimic equivalent *in vivo* responses exactly. *Campylobacter* invasion into CaCo-2 cells show very good correlation to *in vivo* invasive potential in chickens (Hanel et al., 2004) and humans (Everest et al., 1992) but produce limited cytokine responses (MacCallum et al., 2006) whereas HT-29 and T84 produce robust cytokine responses but no good evidence of relevance to *in vivo* invasion responses (MacCallum et al., 2006). In our hands, we could detect cellular invasion in both HT-29 cells and the 8E11 avian cells at a level of 1–3% of the initial inoculum with sufficient robustness to differentiate strains and for consistency over three replicate experiments. Levels of invasion are particularly dependent on time and the starting inoculum, but our results are in keeping with levels of invasion of up to 4% in CaCo-2 cells shown previously (Hanel et al., 2004). This suggests great potential in the avian cell line used in this study for investigating the diversity of *C. jejuni* responses *in vitro*.

This study confirmed the importance of canonical pro- (ERK) and anti-inflammatory (PI 3-Kinase -Akt) pathways for the *C. jejuni*-induced production of IL-8 and IL-10 in human epithelial cells (Watson and Galan, 2005; Li et al., 2011). Furthermore, ERK-dependent *Campylobacter* invasion is also supported by previous studies (Jin et al., 2003; Hu et al., 2006; Samuelson and Konkel, 2013; Samuelson et al., 2013). The importance of these pathways in avian cells is a novel result of this study and confirms that the underlying mechanisms are similar between human and chicken cells. In addition, the significantly higher IL-10 responses in the avian cells suggest that the avian gut may produce IL-10 as a method to tolerate large doses of *Campylobacter* and is supported by previous *in vivo* studies showing that some breeds of chicken produce more ‘regulated’ responses (Humphrey et al., 2014). While the avian cells used here are derived from leghorn chickens it would be interesting to speculate on the breeds used previously (Humphrey et al., 2014). There is evidence using Bayesian structural modeling of *in vivo* responses in chickens that IL-10 profiles are indeed different between breeds and this needs exploring further (Williams et al., 2013; Reid et al., 2016).

Study of *Campylobacter* uptake by endocytic pathways confirmed the requirement of lipid rafts (Lin et al., 2011) in human cells and extended this role to avian epithelial cells. We also found that microfilaments were required for invasion of both HT-29 cells and the avian ones. Interestingly, at least two mechanisms exist as INT407 (Konkel and Cieplak, 1992) and CaCo-2 (Russell and Blake, 1994) cells show microfilaments/microtubules-dependent and -independent mechanisms, respectively, suggesting that the role of cytoskeleton may be strongly cell dependent. Further work into mechanisms of uptake, in the present study, confirmed roles for dynamin and clathrin which has not been documented to date. Interestingly Cdt uptake into cells does involve clathrin coated pits (Thelestam and Frisan, 2004) and may be the mechanisms observed here. We could not confirm a consistent role for caveolins (using filipin and genistein) in the uptake process during this study. Indeed, this is consistent with a recent publication which suggests that *C. jejuni* invasion is independent of caveolins (Konkel et al., 2013).

One interesting consequence of inhibiting endocytic pathways was the concurrent reduction in cell toxicity confirming that *Campylobacter* is responsible for the toxicity and the endocytic inhibitors have negligible effects on cell viability. Another consequence of inhibiting endocytosis was the ‘extra’ effect of inhibiting cytokine production. Previously, De Zoete et al. (2010) established that live *Campylobacter* are very weak stimulators of both human and chicken TLR-2, -4, and -5. In striking contrast, lysed *Campylobacter* induce strong NF-kappaB activation through human TLR1/2/6 and TLR4 and chicken TLR2t2/16 and TLR4 but not via TLR5 of either species (De Zoete et al., 2010). Our results support the concept that ‘invasion’ or ‘internalization’ of some kind is necessary for cytokine production. Indeed, Hickey et al. (2000) also suggest an ‘invasion’ dependent pathway for IL-8 induction.

These results are novel because of the number and diversity of relevant *C. jejuni* strains used on an avian cell line to determine pathogenic mechanisms. However, we realize that there are certain limitations that provide an opportunity to improve our study in future work. Firstly, avian 8E11 cells are derived from small and large intestines of white leghorn chickens and are positive for enterocyte markers, villin, E-cadherin, and cytokeratin. Previous studies have confirmed the importance of broiler breed to the final inflammatory response and suggest that cells from commercial fast and slower growing breeds may be more relevant to study avian gut responses (Kaiser et al., 2016). Indeed, recent technologies point to the precision modeling of chicken intestinal slices (Punyadarsaniya et al., 2015). Secondly, with respect to assay methodology, we recognize that 6 h is probably not the optimal time to measure adhesion and earlier timepoints should be assessed in the future. In addition the gentamicin protection assay is subject to numerous limitations and artifacts as reviewed in detail previously (Friis et al., 2005). Finally, here we focused our attention on using *C. jejuni* strains that define the species, from a variety of sources and sequence types, as published previously (Sheppard et al., 2013). Recent *Campylobacter* outbreaks have highlighted the importance of invasion from the gut (e.g., to the liver) suggesting the importance of focussing on groups of invasive and non-invasive *Campylobacter* in future studies.

## Conclusion

These novel data suggest that avian systems are likely to use similar host defense pathways to humans in response to *Campylobacter* spp. However, the sheer diversity and range of responses suggests that ‘a one strain fits all approach’ to *in vivo* experimental infection would not give meaningful data for the study of *Campylobacter* pathogenesis.

## Author Contributions

DJ was responsible for data generation and analysis across all figures of the manuscript. Design of signaling aspects of the work was developed and overseen by VK. Design of invasion assays was lead by LW. TH and TW were responsible for re-drafting the work and revising it critically for important intellectual content. All authors approved the final submitted draft and had opportunity for editing the document.

## Conflict of Interest Statement

The authors declare that the research was conducted in the absence of any commercial or financial relationships that could be construed as a potential conflict of interest. The reviewer PN and handling Editor declared their shared affiliation.

## Supplementary Material

FIGURE S1 The effect of methyl β-cytodextrin and cytochalasin D on *Campylobacter* invasion. **(A)** HT-29 and **(B)** 8E11 cells were treated with cytochalasin D (5 μM) and methyl-β-cyclodextrin (5 μM) 30 min prior to infection with *C. jejuni* (*n* = 31) for 6 h. Invasion was assessed with each dot representing three biological replicates in one strain. Results are also expressed as mean ± SD of all strains measured. Differences were considered significant if *p* ≤ 0.05.
