# Supplementary figures and images for: The Bacterial Species Campylobacter jejuni Induce Diverse Innate Immune Responses in Human and Avian Intestinal Epithelial Cells
Source: Front Microbiol. 2017 Sep 29;8:1840. doi: 10.3389/fmicb.2017.01840 (PMC5626877; doi:10.3389/fmicb.2017.01840)

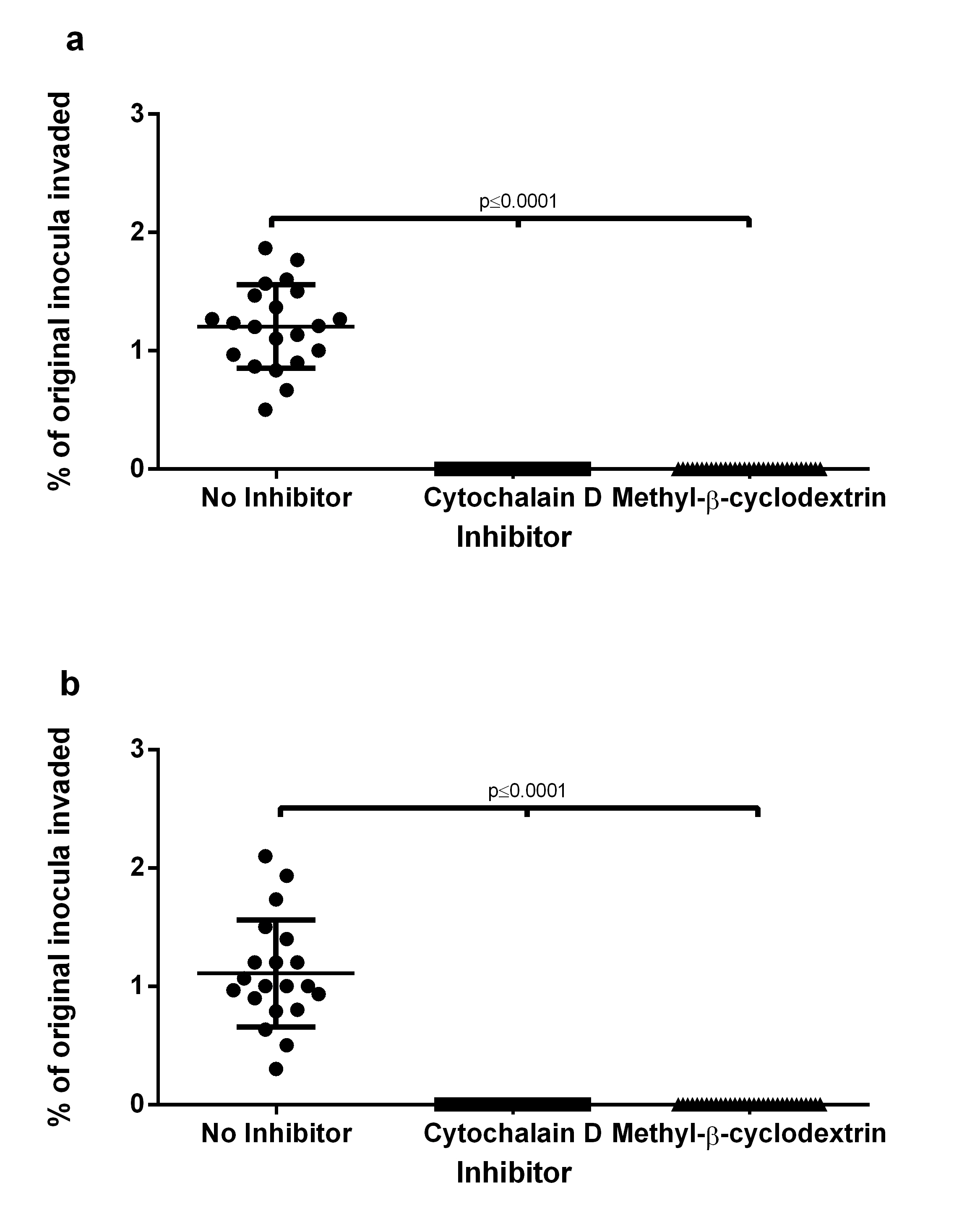

Supplement: FIGURE S1 — The effect of methyl β-cytodextrin and cytochalasin D on Campylobacter invasion. (A) HT-29 and (B) 8E11 cells were treated with cytochalasin D (5 μM) and methyl-β-cyclodextrin (5 μM) 30 min prior to infection with C. jejuni (n = 31) for 6 h. Invasion was assessed with each dot representing three biological replicates in one strain. Results are also expressed as mean ± SD of all strains measured. Differences were considered significant if p ≤ 0.05. [file Image_1.jpg]
